# Using focus groups to inform a peer health navigator service for people who are transgender and gender diverse in Saskatchewan, Canada

**DOI:** 10.1111/hex.14022

**Published:** 2024-03-25

**Authors:** Gwen Rose, Michelle McCarron, Mel Reid, T. Fayant‐McLeod, Emily Gulka, James Young, Megan Clark, Stéphanie J. Madill

**Affiliations:** ^1^ School of Rehabilitation Science, College of Medicine University of Saskatchewan Saskatoon Canada; ^2^ Saskatchewan Health Authority Regina Canada; ^3^ Community Researcher Regina Canada; ^4^ Community Researcher Toronto Canada; ^5^ Academic Family Medicine, College of Medicine University of Saskatchewan Saskatoon Canada

**Keywords:** discrimination, healthcare access, healthcare navigators, qualitative research, Saskatchewan, Canada, transgender

## Abstract

**Background:**

This study investigated healthcare access and quality for people who are transgender and gender‐diverse (PTGD) in Saskatchewan (SK), Canada, to inform a larger project that was piloting two peer health navigators for PTGD.

**Methods:**

Two online focus groups were held. Nineteen participants were recruited to represent a broad range in age, gender and location in SK. Transcripts of the focus groups were analyzed using a thematic approach.

**Results:**

The core theme that was identified was participants' desire for culturally safe healthcare. This core theme had two component themes: (1) systemic healthcare factors and (2) individual healthcare provider (HCP) factors. The healthcare system primarily acted as a barrier to culturally safe healthcare. HCPs could be either barriers or facilitators of culturally safe care; however, negative experiences outweighed positive ones.

**Conclusions:**

PTGD in SK face discrimination, with delays and barriers to care at all levels of the healthcare system. Peer health navigators can address some of these discrepancies; however, greater support is required for PTGD to be able to access culturally safe healthcare.

**Patient or Public Contribution:**

People with lived experience/PTGD were involved in all stages of this project. They were included on the team as community researchers and co‐developed the research project, conducted the focus groups, participated in the analyses and are co‐authors. As well, both navigators and all the participants in the focus groups were also PTGD.

## 1 INTRODUCTION

People who are transgender and gender diverse (PTGD) experience many barriers when accessing healthcare. The 2021 Canadian Census found that 0.33% of Canadians aged 15+ identified as ‘transgender’ or ‘non‐binary’.^1^ A 2019 Canadian survey of PTGD found that 44.4% reported a past‐year unmet healthcare need, eight times the rate of the general population.^2^ Additionally, nationally 40.7% of respondents were waiting for gender‐affirming care, with a 43.6% adjusted predicted probability of being on a waitlist for gender‐affirming care in Saskatchewan (SK), Canada.^2^ Among SK respondents, 86.5% had a primary care provider, but only 54.4% felt comfortable discussing trans health issues with them.^2^ This was echoed in a 2022 survey of primary care providers in SK, which found that 95.8% of respondents felt comfortable providing general healthcare to PTGD (*p* = .072), but only 30.3% felt comfortable providing transition‐related care (*p* = .290).^3^

Most research on the healthcare experiences of PTGD has studied people living in large urban centres or in places with different funding systems.^4^, ^5^ In a Canadian context, only the aforementioned studies by Scheim et al. and Christopherson et al. addressed the experiences of PTGD in SK. Most other studies are from Ontario, Canada's most populous province; The Trans PULSE project in particular investigated many aspects of life and health for PTGD in Ontario,^6^, ^7^, ^8^, ^9^ which differs from SK by having a much more concentrated urban population, public transit to rural communities in the London‐Toronto‐Kingston‐Ottawa corridor, as well as a different health system since health is a provincial responsibility in Canada. Other Canadian studies of PTGD and their health have also been limited to select provinces, including British Columbia^10^ and Nova Scotia^11^ or even just the city of Vancouver, British Columbia.^12^ Other studies have not mentioned their location, but have also had a narrow geographical focus, for example, Bell and Purkey's^13^ study, which analyzed the primary care experiences of PTGD in Kingston, Ontario. Finally, some researchers presented their work as addressing the healthcare experiences of PTGD in Canada in general, but had participants largely from large urban centres, such as MacKinnon et al.'s^14^ study, which recruited participants largely from the Greater Toronto Area, Ontario's (and Canada's) largest urban centre.

SK is distinct in several ways from other places where healthcare for PTGD has been studied. It is a large province with a small population (2.0 persons/km^2^).^15^ 66% of the population lives in towns or cities, which is 18% lower than the national average.^15^ Although SK has universal government health insurance, this population distribution concentrates healthcare services in the south and leaves the north with a paucity of services.^16^

The Trans Research and Navigation Saskatchewan (TRANS) Project was developed to fill this research gap. It is a collaborative, community‐driven research group based at the University of SK. The project is rooted in the Canadian Professional Association for Transgender Health (CPATH)'s ethical guidelines.^17^ In keeping with these guidelines, the researchers were a combination of academic researchers, healthcare providers (HCPs) and PTGD. All research activities included PTGD as decision makers. The overall purpose of this project was to pilot and evaluate peer health navigators for PTGD, filling a community‐identified need for improved access to affirming healthcare. Two peer health navigators provided services for 1‐year (April 2021‐April 2022); both were PTGD with experience working in the healthcare system. They supported clients (PTGD and their support people) and HCPs. Services were provided in compliance with COVID‐19 public health regulations; most were provided by phone, text message or email.

This paper concentrates on an iterative activity used to inform and refine the navigators' duties: focus groups to learn about the healthcare experiences of PTGD who had navigated the system themselves. Based on a combination of team members' lived experience, prior research,^3^ and the navigators' early observations, we were aware of some structural barriers to affirming care. However, we still had questions about their effects on individuals. The affective meaning of these experiences also interested us, as they could either add to or lessen the burden of accessing care. Exploring the experiences of people who had not used the navigators' services allowed us to gain insight into experiences that were otherwise invisible to the navigators and to improve access to healthcare for PTGD in SK.

## 2 MATERIALS AND METHODS

### 2.1 Study design

We conducted two focus groups in September 2021, one with participants from Saskatoon/northern SK and the other from Regina/southern SK. Participants discussed their experiences with healthcare in the previous 18 months. Participants were included if they self‐identified as PTGD. The study was advertised via LGBTQ+ organizations' social media and posters in family medicine clinics. A $50 honorarium was provided. We selected participants to represent a diversity in geographic location, age, and gender, with priority given to geographic location because of the limited to access healthcare in northern SK.^16^ Participants could bring a support person with them if they wished, though no one did. Groups were limited to 12 participants each.

Due to COVID‐19, the focus groups were conducted via videoconferencing (WebEx, through the University of Saskatchewan; https://www.webex.com/). Sessions were approximately 90 min each and were facilitated by the same research assistant (G. R.), using a structured interview guide. One researcher (S. J. M.) attended the sessions to take notes, while another research assistant (M. R.) attended as a trained support person. The facilitator posed the following questions: (1) Have you been able to connect with the healthcare you needed? (2) What is your perception of HCPs' attitudes towards PTGD? (3) What is your impression of healthcare services for transition‐related care in SK? and (4) What else should we have asked? Everyone who wished to speak was given a chance to speak to the question before people were invited to speak again. Responses posted in the WebEx chat were included in the analyses.

Each session was recorded, and data stored by the Canadian Hub for Applied and Social Research. Sessions were transcribed verbatim by the research team, and names were anonymized through randomized initials. Excerpted quotes in the results have been edited for clarity.

Six researchers (G. R., M. R., M. M., T. F.‐M., J. Y., E. G.) completed a thematic analysis of the transcripts using NVivo software (https://www.qsrinternational.com/nvivo-qualitative-data-analysis-software/home). Three researchers worked on each transcript, to reduce the workload on the community researchers (all of whom are PTGD), and to avoid identifying any participants. Through consensus, a single list of codes was created for each transcript, with similar terms being amalgamated. Then all six researchers met and, through consensus, combined the two lists of codes into a single list—again, amalgamating similar terms—then grouped the codes into emergent themes, while repeatedly referring back to the transcripts to ensure that the original meaning was retained. For example, instances of HCPs using the wrong pronouns were coded as ‘misgendering’, which became part of the theme ‘HCP level factors’. As being misgendered was also a negative experience, the same text was coded as ‘negative emotions’, which could be cross‐referenced as needed.

Throughout the analysis process, deidentified and summarized results were reported verbally to the peer navigators at regular meetings. Ways that the navigators could respond to the focus groups' concerns, bolster the areas of strength in the system and implement their recommendations were discussed at the meetings.

This study was approved by the University of Saskatchewan Behavioural Research Ethics Board (Beh‐1897) and all participants provided consent.

## 3 RESULTS

### 3.1 Demographics

We had exactly 24 initial respondents who met the inclusion criteria, so all were invited to participate. Of these 24, 19 participated: four ceased responding after initially expressing interest, and another was unable to attend at the last minute. Demographic information for the 19 participants (see Table 1) has been summarized across the focus groups to protect the participants' anonymity.

**Table 1:** Participant characteristics.

	Number of participants
Age, years old	
<18	5
18–30	6
>30	8
Sex assigned at birth	
Male	5
Female	13
Intersex	1
Pronouns^a^	
He/him	9
She/her	5
They/them	10
Identity (self‐described)^a^	
Trans man	10
Trans woman	5
Intersex	1
Two spirit	1
Nonbinary/gender‐nonconforming	9
Location	
Urban	16
Rural	3
Healthcare access frequency	
Weekly	3
Biweekly	6
Monthly	3
<Once a month	7

^a^ Some participants used multiple pronouns and/or described their identity using multiple terms.

#### 3.1.1 The focus group process

All participants spoke at least once and most contributed throughout. Engagement in the discussions was high, and participants built on one another's responses. While negative experiences were reported more frequently, and elicited longer discussions than positive ones, there was still a large number of both types of experiences reported, by many participants. Only a general suggestion that we were interested in both positive and negative experiences was given at the outset of the sessions.

### 3.2 Themes

Both focus groups demonstrated the same themes, therefore researchers considered data saturation to have been achieved. One overarching theme emerged: the need for culturally competent healthcare for PTGD. It was supported by two‐component themes: systemic healthcare factors and HCP factors, that is, how PTGD accessed and experienced healthcare was linked to both the organization and processes of the healthcare system, and to experiences with individual HCPs.

#### 3.2.1 Overarching theme: Need for culturally safe healthcare

The need for culturally safe healthcare for PTGD was conceptualized as an overarching theme because access to it was dependent upon the other two themes (systemic factors may facilitate or impede culturally safe care, while individual HCPs may provide it, or not). This need was represented in emotionally charged responses that, when positive, demonstrated its benefits, and, when negative, demonstrated the harm that the system or HCPs can enact. T. E. provided an example of the latter:

I went to go see a doctor and he was supposed to be trans‐positive […] it was supposed to be a good experience that could be a safe kind of environment. […] He seemed fine when we were in the actual room […] but, then I had to get some paperwork for my insurance company, and I ended up seeing his notes that he had written and a letter to [my family physician], and through the entire document [he] was misgendering me, and, it was just, I was so excited that I was on the way to seeing these specialists for potentially getting surgeries and then to see this, and it basically was a punch in the gut […] I literally sat in the car and I cried.

Here, we see that when an HCP failed to be culturally safe, it not only undermined the positive feelings of getting a referral for gender‐affirming care but, as T. E.'s words show, created negative feelings and even a negative physical reaction.

The importance of culturally safe care was also reflected in positive experiences, where HCPs affirmed PTGD identities and provided gender‐affirming care. As C. D. related:

I ran out of testosterone, and [experienced] a hurried rush trying to find [more] before I got the last shot out of the bottle. [Thanks to] the pharmacy that my spouse and I go to, they were able to connect me with […] three different people that could have helped: one was an endocrinologist who wouldn't take me because I wasn't a patient yet, and had no health card [CD was an immigrant and not yet covered by provincial health insurance]. But we ended up finding that Dr. I at [a medical clinic] was extremely helpful […] he looked at my vial and said, ‘you've been on this for how long’ and I told him my entire history, and he's like ‘ok, then I can refill this dosage, give you extra dosage, just in case something happens to [the] vials’. So… I was very lucky that, just by reaching out, even though I was a bit in a sheer panic, that I was able to find the help that I needed.

In this case, the HCP recognized the systemic barrier to the participant's care and overcame it, choosing to refill the prescription even though C. D. lacked insurance, thereby allowing them to continue their gender‐affirming care without interruption. Nor was C. D.'s gender identity questioned: by providing trans‐specific care *and* affirming CD's identity, this HCP provided care which relieved the ‘panic’ that C. D. felt.

The word ‘lucky’ came up in many positive accounts, including C. D.'s, reflecting participants' *expectations* that culturally safe care would be difficult to find. For example, D. G. said that when he came out, he was ‘lucky’ that his ‘family doctor [knew who] to send [him] to’ for gender‐affirming care, and within ‘four months’ he had secured a testosterone prescription from the referral. D. G. felt ‘lucky’ simply because his family doctor knew where to refer him for gender‐affirming care, and he ‘didn't have to go somewhere and look for my own’. Beyond these feelings of being ‘lucky’, D. G.'s story conveys that having an HCP who is knowledgeable about the processes for receiving gender‐affirming care *helped him receive care faster*. Even though many family doctors in SK choose to refer PTGD to physicians willing to provide gender‐affirming care, which creates barriers; a culturally safe provider, even one who is not willing to provide gender‐affirming care, can save PTGD time waiting for care by referring immediately. D. G.'s success in getting a referral contrasts with B. T.'s experience after moving to SK from another province; she described hoping to find ‘a family doctor [and] an endo[crinologist]’ and said that she had ‘not yet succeeded in’ accessing ‘any of those’. B. T. accessed her existing prescriptions for hormones only from walk‐in clinics, which is a precarious position. This accords with the perceptions of other participants that finding affirming care quickly and easily was uncommon and unexpected (i.e., ‘lucky’). These examples, among others, demonstrate the importance of culturally safe care for PTGD.

#### 3.2.2 Component theme: Healthcare system factors

##### Barriers

Systemic factors included long wait times, a lack of HCPs (particularly in rural areas), financial concerns, and administrative processes. Unlike experiences with individual HCPs, systemic factors were rarely associated with positive experiences, and the positive aspect of such experiences was not due to the system. For example, after ‘two months’ of care, G. T.'s family physician

asked if it was in my plan to get top surgery, so she got me on the waiting list to see Dr. N. That was the longest waiting list […] a year and a half to two years. But after going and doing referral appointments and stuff like that, I ended up getting top surgery, and […] I'm now seventeen, and I am pretty much where I want to be with my transition.

Here, wait times are depicted as long, but the overall experience was positive, because G. T. was able to complete their personal transition goals by a young age. We noted the strong feeling of connection between G. T. and the culturally safe HCP, who, by asking the right question about G. T.'s plans for top surgery, was able to reduce G. T.'s emotional stress and wait time by getting the process started immediately. This example—among others—clearly demonstrates the connection between the theme of systemic barriers and the overarching theme of the importance of culturally safe care (and HCPs).

Wait times were reported as a barrier by *all* participants and were perceived as related to a lack of culturally safe HCPs—because the lack of HCPs meant longer wait times or difficulty finding an HCP at all. Participants wanted affirming primary HCPs; those who had them expressed emotions such as gratitude and relief, whereas those who were either still looking for or were on wait lists expressed emotions such as anger, frustration and resignation. Whether or not they had affirming HCPs themselves, all shared concern over the limited number of culturally safe HCPs in SK and the long wait times to see them. For example, G. T. said:

I went to see Dr. H [a trans‐affirming family physician] and she had a pretty long waiting list, but I got [in] after maybe like a year of waiting. […] later she asked if it was in my plan to get top surgery, so she got me on the waiting list to see Dr. S [plastic surgeon], and that was the longest waiting list, ‘cause I was maybe on it for about a year and a half to two years.

G. T. went on to explain that delaying transitioning

affects your mental health greatly. And then, that also ties into the mental healthcare system, which often isn't accessible either, and there's often long wait times for free mental healthcare. Because a lot of the time it isn't covered by [public] insurance […] it leads into this big jumble where transition healthcare is backed up, and there's a long wait line, but then mental healthcare is also backed up and there's a long wait line, because people can't transition.

The participant clearly outlines the connection between long wait times for transition‐related healthcare, and mental health concerns, which, while not elaborated on, implied stress, anxiety, and/or depression.

While wait times resulted in unmet needs for many participants, for rural participants the increased distance from culturally safe HCPs exacerbated existing concerns with wait times because access to care required extensive travel. I. H. related that when he lived in Northern SK,

getting access to care from [there] was an ordeal. I didn't have any access up there to anybody, you know, I had to get on a waitlist to see the right doctors in the city, and then all of my appointments were [in Saskatoon, approximately 300 km away]. I had [to] go to Regina for some, I had to go actually into Alberta for others. And getting that for my trans stuff, getting access for that kind of stuff was really hard and there [were] a lot of cancelled appointments, delays, and you know, I would have a date set, and then my appointment would get pushed back three months or, I would get a notification that I'd have to make the phone call to reschedule and stuff like that.

The lack of culturally safe providers outside of SK's limited urban settings meant hours of travel with associated expenses. These were all systemic issues that interfered with I. H.'s ability to access timely gender‐affirming care and which highlighted the additional barriers facing PTGD living in SK's large rural areas.

Administrative and financial concerns related to which procedures are covered by provincial health insurance were another systemic issue that contributed to poor access to care for PTGD. SK has a single‐payer healthcare system, but it does not cover procedures, like laser hair removal, that are considered cosmetic, and while gender‐affirming surgery is covered, only transmasculine chest surgery and removal of reproductive organs are available in the province.^18^ Provincial insurance pays the surgical costs and postoperative hospice stays for genital surgery at the GRS clinic in Montreal; however, it does not pay for any travel costs. C. D. and C. M., a couple on the same WebEx account, noted that ‘surgeries […] related to genitalia’ require a ‘flight to Montreal […] with wait times at those clinics’ being long as well, ‘three years’ in the words of these participants. As C. D. and C. M. pointed out, ‘there's a price tag on being trans’.

Participants also highlighted that the referral process for out‐of‐province gender‐affirming surgery is a barrier, because approval requires referral letters from two approved physicians, one of whom must be a psychiatrist.^19^ Participants explained that this burden broke down into several categories. First, the approved HCPs have their own waitlists. Second, most participants did not have a pre‐existing relationship with the approved HCPs, so faced anxiety and emotional stress as they anticipated building rapport and convincing a new provider that they qualified for the needed procedure. Furthermore, the small number of approved HCPs have their practices in the province's two major cities, or outside SK (Saskatchewan, n.d.), so PTGD who live in rural areas also have the travel issues discussed above. C. D. highlighted these combined burdens, noting that because PTGD

have to have letters signed off by these specific doctors […] when I'm looking at the list, I'm seeing I'm going to have to go to Saskatoon to get a letter from a [psychiatrist] who would have met me once, maybe? Because I'm not in Saskatoon, so it's not like it's going to be my regular therapist.

We see that participants were keenly aware of the bureaucratic processes involved in accessing trans care in SK, and perceive both the processes themselves, along with the wait times, as significant barriers to care.

Another barrier to receiving affirming care was changing one's name and gender marker in SK's vital statistics system. Many participants reported being repeatedly misgendered and dead‐named by HCPs until they made the changes, even when they asked HCPs to use their correct names and pronouns. E. S. related that ‘changing my name with eHealth’ was ‘a process—they rejected me three times, because of rules that aren't on their forms […] or on the site, but what can you do?’ clearly expressing frustration and resignation with a system that created barriers by being overly complicated. Frustration with these processes and lack of clear instruction was echoed by other participants.

#### 3.2.3 Component theme: HCP factors

Finally, experiences with HCPs predominated both focus group discussions, and while they interacted with systemic factors, they also clearly constituted their own theme, as, regardless of the formal structure of the healthcare system, interactions with individual HCPs were meaningful in and of themselves.

#### 3.2.4 Facilitators

Positive experiences with HCPs and staff were particularly meaningful for participants. Even when they were not able to provide gender‐affirming care directly, their efforts to make participants feel welcome and affirmed were clearly important. For example, as G. T. related,

My family doctor, he's probably not as well educated on it, but he's […] understanding, and him [sic] and his receptionist, they're always [saying] ‘just let us know when you change your name and we can change it legally, but for now we'll just call you GT and make sure that your legal name is still on the paperwork, just in case’. So, they're really understanding…

The HCPs' understanding of G. T.'s identity, and willingness to implement this within their practice, created positive healthcare experiences.

Participants described HCPs who demonstrated an understanding of trans‐specific healthcare needs alongside accepting attitudes as ‘knowledgeable’, which to them meant both well‐informed regarding gender diversity more broadly as well as the specific healthcare needs of PTGD. Although participants identified this level of knowledge as basic, due to a predominance of prior negative experiences, they expressed surprise when they encountered it. For example, I. H. recalled being ‘pleasantly surprised’ when a new HCP ‘knew exactly what it was that I needed, why I need it, [and] how I need it, and everything. She talked me through the whole process’. describing this HCP as ‘awesome’. The scenarios in which participants previously described feeling ‘lucky’ are also examples of this. E. S. related another positive experience:

I had one meeting with [a family physician], because I'm considering starting testosterone, and she was very clear with the pros and cons of it […] Dr. K was an amazing person to talk to […] she talked through it with me, and she made me more comfortable with the decision of not doing hormones because she […] showed knowledge, and she showed it in a respectful way. She didn't come off as a know‐it‐all doctor, I guess, not someone who pretends that they know more about your life than you do, when it comes to transition, which I think, I was really grateful for.

Here, the participant felt affirmed when the HCP demonstrated knowledge and experience regarding gender‐affirming care and respected both them as a person and their ability to arrive at the appropriate decision for themself; the participant was ‘grateful’ for this positive experience and high quality of care.

#### 3.2.5 Barriers

Negative experiences were, of course, also present in both focus groups, as participants shared their experiences with HCPs who had limited understanding of trans identities or healthcare needs, and who displayed discomfort interacting with PTGD, resulting in negative and even transphobic experiences. T. E.'s experience presented earlier clearly illustrates this, as the HCP's refusal to affirm T. E.'s gender in referral documents provoked an intensely negative emotional response. Participants had a prolonged discussion with HCPs who were perceived as *not* culturally safe because they did not have even a basic understanding of trans identities. N. C. summarized the participants' general sentiment by commenting that ‘it's insane how much trans people are expected to educate others when it shouldn't be our job’. Other examples included ill‐informed HCPs who conflated gender identity and sexuality: N. C. related that on multiple occasions, after disclosing a nonbinary gender identity, HCPs would ask ‘extremely personal questions about intercourse and stuff, some of them got really bad. And […] not only am I a minor, but I shouldn't have to explain that to someone’. This prompted E. S. to recount: ‘that was my experience with my family doctor. I definitely got asked a lot of questions about my sex life, and my sexual orientation’. These examples collectively serve to demonstrate the widespread lack of culturally safe HCPs, and the impact of the interactions with such HCPs. These experiences were frustrating, emotionally stressful, and associated with low standards of care.

I. H.'s experience at a walk‐in clinic serves as a useful summary of all three themes. Still discussing the difficulty of accessing care in rural locations, I. H. noted that when travelling for work, he needed to go to walk‐in clinics, where sometimes, in his words, HCPs ‘are not very trans‐friendly’. In this instance, I. H. went to a walk‐in clinic for

a personal reason down‐below, and [the physician] took two seconds to look at it. And he actually got the diagnosis wrong, 'cause he just did not want to deal with anything that was, you know, down there, and I mean, I don't want to deal with him dealing with it either, but […] I thought he would be more professional about it, he just—took two seconds, looked, and was done. And after that, couldn't make eye contact with me, just didn't want to deal with me.

I. H. was acutely aware that his genitals were incongruent with the physician's expectations and that this was related to a lack of professionalism. Here a systemic factor—the lack of culturally safe HCPs in a rural area—intersects with an HCP factor. With no culturally safe HCP available, the participant had to see an HCP who provided poor care because of his reluctance to engage with a transgender body. PTGD can perceive an HCP's insensitive or disgusted reaction to their identity and/or body: I. H. stated that, given the HCP's obviously transphobic reaction, he did not ‘want to deal with [the physician] dealing with it either’, yet he had no other option. In instances like this, systemic and HCP factors interact to create emotionally unsafe, substandard care for PTGD: this in turn informs and confirms the overarching theme of the importance of culturally safe care.

### 3.3 Impact on navigator programme

The findings from the focus groups informed the navigators' work in the following ways. Participants' emphasis on cultural safety reinforced the navigators' ethos of person centredness and the importance of building supportive relationships with clients. To address systemic barriers, the navigators became notaries public to facilitate the process of name and gender marker changes, and they wrote letters and put out calls on social media requesting that more family physicians take on basic trans healthcare. To improve interactions with HCPs, they provided group in‐service education about trans identities. They also provided information to HCPs about trans healthcare and processes, such as surgical referrals, provided links to Canadian trans healthcare guidelines, for example, Rainbow Health,^20^ and made connections to mentors.

## 4 DISCUSSION

We identified an over‐arching theme in the participants' responses: the need for competent, culturally safe healthcare for PTGD to ensure that they received care in a way that affirmed their gender and created positive emotions. This theme was composed of two themes and their interactions: (1) Healthcare system factors: systemic barriers that arose from the healthcare system, provincial health insurance or other institutional policies; and (2) HCP factors: facilitators (knowledgeable and respectful HCPs) and barriers (HCPs who were not comfortable with PTGD, or who invalidated trans identities) to receiving affirming care from individual HCPs. Across the three themes, facilitators corresponded with strong positive emotions and experiences. Conversely, encountering barriers was a consistently negative experience.

### 4.1 Culturally safe healthcare for PTGD

The emotionally charged language used by participants to describe instances of culturally safe healthcare, or healthcare that was distinctly unsafe, demonstrated that they felt these experiences in their bodies and that the experiences were shaped by their expectations of the experience, which were in turn informed by their prior healthcare experiences. This was consistent with research from SK that found that people who are lesbian, gay, bisexual, transgender and queer expect discrimination from HCPs based on previous experiences of stigma or discrimination from physicians,^21^ and demonstrated the concept of embodiment, which maintains that people are simultaneously and inseparably biological and social entities.^22^ Participants consistently used stronger emotional language and reported greater effects when describing culturally unsafe healthcare encounters. This is consistent with the negativity bias that explains how people tend to experience negative experiences as more emotionally salient than positive ones.^23^ Both embodiment and the negativity bias have implications for healthcare practice through the other two themes: systemic healthcare factors and individual HCP factors.

### 4.2 Systemic healthcare factors

Participants experienced the healthcare system mostly as a barrier. Access to healthcare was facilitated by individual HCPs either working around the system or knowing the system well enough that they could direct the participant to needed care. The reported lack of affirming HCPs is consistent with a survey of primary HCPs in SK, which found that only 30.3% felt comfortable providing transition‐related care to PTGD.^3^ The poorer access to care rurally was consistent with research from several locations including Tennessee, United States and Ontario, Canada.^24^, ^25^

The referral process for out‐of‐province gender‐affirming surgery was particularly problematic. PTGD who need referrals typically do not have established relationships with the province's recognized authorities and are frequently hesitant to engage with new HCPs due to negative expectations and/or feeling that such assessments are exercises in gatekeeping wherein they must ‘prove’ their trans identity.^25^ This is counter to the World Professional Association for Transgender Health's (WPATH's) Standards of Care which recommend a single formal assessment for PTGD seeking gender‐affirming treatments from the individual's regular HCP.^26^ WPATH stresses that providing gender‐affirming care in a timely fashion has positive mental health outcomes; in contrast, SK's system increases wait times, which in turn has a negative impact on the well‐being of PTGD.

Several other systemic barriers were frequently mentioned, including costs related to changing gender markers in eHealth, travel for out‐of‐province procedures, and for healthcare services not covered by provincial insurance. However, research suggests that procedures such as laser hair removal can significantly alleviate gender dysphoria.^27^ Costs, in the form of co‐payments and a lack of insurance coverage, were also found to be a barrier to care for PTGD in New Zealand^28^ and the United States.^29^ Financial stress can compound the emotional stress of having to wait to access or being unable to access healthcare.

The healthcare system's effect on the participants' wellbeing loops back into embodiment, as whether the participants' physical and emotional needs were met was tied to the social structures imposed by the healthcare system. Overall, the system was neutral or impeded people's needs, which fed their negativity bias when approaching future healthcare encounters.

### 4.3 HCP factors

Participants experienced individual HCPs as gatekeepers of affirming healthcare. Again, embodiment was clear in participants' language and the negativity bias was evident through participants expecting to be treated badly by HCPs, using the most emotional language when discussing interactions that went poorly and by using language such as ‘lucky’ and ‘pleasantly surprised’ to describe affirming interactions.

Our findings are consistent with Christopherson et al.'s^3^ findings that many SK primary HCPs feel unprepared to provide trans healthcare and Schwab et al.'s^21^ findings that HCPs are frequently uncomfortable with trans identities. Research from New Zealand also found that PTGD feel they must educate their HCPs about their identities and healthcare needs.^28^ Our participants asserted that HCPs need more education on the healthcare needs of PTGD, which is supported by Burgwal et al.'s^30^ conclusions that providing HCPs with cultural and medical training about trans identities and healthcare needs increased their competence in treating PTGD. This would benefit the mental health of PTGD, along with their physical health.

Participants' focus on positive experiences echoes findings from focus groups in Boston and New York City, United States^31^ where participants requested that researchers focus on their strengths and resiliency, and not just the disparities they faced. The language our participants used to express how these positive experiences felt demonstrated that they were important in bolstering their sense of self and increasing their resilience, for example, finding long wait times for surgery more tolerable when they were supported by affirming HCPs.

### 4.4 Strengths and limitations

Strengths of this research include our commitment to CPATH's ethical guidelines^17^ by engaging directly with PTGD throughout our research. Our research team, while led by cisgender academic researchers, included meaningful contributions from PTGD throughout, from research conception to authorship. We chose to work with a large data analysis team to avoid burdening our community researchers,^32^ and to ensure that we centred the perspectives of PTGD.

One limitation of the research was the relatively young age of our participants; other research estimated that the average age of PTGD in SK is 42.5 years.^33^ This may have been a result of our recruitment strategy. Another consequence of our recruitment strategy was that we had few participants from rural and northern SK. This may have resulted in missing nuances in the healthcare experiences of older and rural PTGD. More participants identified as transmasculine than transfeminine; however, this ratio is consistent with previous research.^33^

Employing focus groups, instead of one‐on‐one interviews, may have made some participants less comfortable and limited their contributions to the discussion. Our research group had extensive discussions about how to structure this part of the study. While we recognized that a group setting might make some people reticent to share, we decided to use focus groups for three reasons: sharing *healthcare* experiences with acquaintances at social gatherings is a very common part of trans culture that is considered almost a responsibility to protect others from culturally unsafe HCPs^34^; in these informal settings, one person's account tends to remind others of similar experiences they have had and we observed the same effect during the focus groups, and given the limited resources available with our grant, focus groups allowed us to gather the experiences of more people than we could with individual interviews.

### 4.5 Future directions

In direct continuation from this study, future research should investigate the experiences of PTGD who are older than 30, who live in rural and remote areas and who are racialized to determine how these identities intersect with gender when accessing healthcare. Specific to the SK context, research should consider the effects of being Two‐Spirit, a distinct identity category unique to Indigenous peoples.

The navigators continue to work under a community organization with interim funding from the SK government and a federal grant. These findings are being used to advocate for long‐term funding.

## 5 CONCLUSION

Overall, there is a lack of culturally safe, gender‐affirming care for PTGD in SK, although pockets of good care do exist. At the system level, participants identified numerous barriers to care, including, but not limited to, restrictions on certain types of transition‐related care related to provincial insurance coverage and referral processes, difficulty with changing gender markers, and a lack of HCPs willing to care for PTGD. At the HCP level, barriers were related to a lack of affirming HCPs and a need for improved education concerning trans health. These themes indicate significant healthcare inequities for PTGD in SK, some of which could be addressed by the navigators, including teaching HCPs to provide basic healthcare to PTGD, so they no longer need to refer to other HCPs. However, other barriers were beyond the navigators' abilities and must be addressed through reforming the provincial health insurance system and increasing the number of HCPs overall. By assuring PTGD that competent healthcare is available, HCPs meet their ethical obligation to improve quality of life through demonstrating compassion and integrity.

## AUTHOR CONTRIBUTIONS

**Gwen Rose**: Project administration; formal analysis; writing—original draft; writing—review and editing; resources; data curation. **Michelle McCarron**: Formal analysis; writing—review and editing; methodology. **Mel Reid**: Formal analysis; writing—review and editing; data curation. **T. Fayant‐McLeod**: Formal analysis. **Emily Gulka**: Formal analysis. **James Young**: Formal analysis. **Megan Clark**: Conceptualization; methodology; data curation; supervision; funding acquisition; project administration; writing—review and editing. **Stéphanie J. Madill**: Conceptualization; data curation; methodology; supervision; project administration; writing—review and editing; funding acquisition.

## CONFLICT OF INTEREST STATEMENT

The authors declare no conflict of interest.

## Data Availability

Research data are not shared.
